# 3DCellAtlas Meristem: a tool for the global cellular annotation of shoot apical meristems

**DOI:** 10.1186/s13007-019-0413-0

**Published:** 2019-04-04

**Authors:** Thomas Montenegro-Johnson, Soeren Strauss, Matthew D. B. Jackson, Liam Walker, Richard S. Smith, George W. Bassel

**Affiliations:** 1 School of Mathematics, University of Birmingham, Birmingham, B15 2TT UK; 2 Max Planck Institute for Plant Breeding Research, 50829 Cologne, Germany; 3 School of Biosciences, College of Life and Environmental and Life Sciences, University of Birmingham, Edgbaston, Birmingham, B15 2TT UK; 4 School of Life Sciences, University of Warwick, Coventry, CV4 7AL UK

**Keywords:** Shoot apical meristem, Cell atlas, Cell annotation, 3D imaging, *Arabidopsis*, Tomato, Cell segmentation, Organ growth

## Abstract

Modern imaging approaches enable the acquisition of 3D and 4D datasets capturing plant organ development at cellular resolution. Computational analyses of these data enable the digitization and analysis of individual cells. In order to fully harness the information encoded within these datasets, annotation of the cell types within organs may be performed. This enables data points to be placed within the context of their position and identity, and for equivalent cell types to be compared between samples. The shoot apical meristem (SAM) in plants is the apical stem cell niche from which all above ground organs are derived. We developed 3DCellAtlas Meristem which enables the complete cellular annotation of all cells within the SAM with up to 96% accuracy across all cell types in *Arabidopsis* and 99% accuracy in tomato SAMs. Successive layers of cells are identified along with the central stem cells, boundary regions, and layers within developing primordia. Geometric analyses provide insight into the morphogenetic process that occurs during these developmental processes. Coupling these digital analyses with reporter expression will enable multidimensional analyses to be performed at single cell resolution. This provides a rapid and robust means to perform comprehensive cellular annotation of plant SAMs and digital single cell analyses, including cell geometry and gene expression. This fills a key gap in our ability to analyse and understand complex multicellular biology in the apical plant stem cell niche and paves the way for digital cellular atlases and analyses.

**Electronic supplementary material:**

The online version of this article (10.1186/s13007-019-0413-0) contains supplementary material, which is available to authorized users.

## Background

The ability to accurately capture, quantify and compare phenotypes across scales is central to understanding genome function, and establishing genotype–phenotype relationships. In plants this has been largely examined at macroscopic levels [12, 15].

Due to advances in sample preparation [7, 8, 33, 34] and microscopy [22], full 3D and 4D cellular resolution imaging of whole plant organs are now routinely being generated [2, 16, 27, 29, 37, 39]. The computational analysis of these image datasets can provide outputs that can bridge the organ, cellular and molecular scales [6, 9, 13]. Plant developmental biology has made use of many of these techniques to understand the basis of growth and development, both in terms of cell growth [2] and cell division and lineage tracking [17, 24, 37, 39].

With the continued generation of these informative organ-wide 3D cellular datasets comes the need to extract biologically meaningful information. Similar to gene expression datasets, quantitative 3D cellular images require annotation in order to contextualize the data obtained into cell identity and position [26]. The inability to perform cellular annotation represents an obstacle in the ability to analyse these quantitative image datasets, to extract their key biologically significant features through the functional annotation of data points (cells), and to identify equivalent data points between different samples. In this instance, individual cells and their properties can be treated as quantitative data points within the complex structure of a plant organ. The annotation of cells within organs based on their identity and/or position enables their context within an organ to be established, and their associated data to be analysed accordingly.

We previously developed a computational pipeline named 3DCellAtlas which performs both cellular annotation and position identification within radially symmetric organs, enabling digital single cell analyses [28]. Not all plant organs are radially symmetric, making this approach limited to those which share this symmetry.

The shoot apical meristem (SAM) in plants is the apical stem cell niche from which all above ground organs develop, and is the subject of intensive study across numerous labs [4, 18, 37]. Both 3D and 4D cellular resolution imaging of the SAM is now being routinely performed by a variety of labs [3, 11, 21, 23, 37], with software to perform automated cell lineage tracking [16] and registration [27] having been developed. These represent rich dynamic datasets which have yielded novel insights into plant stem cell biology and organ development.

Here we report the development of a software package called 3DCellAtlas Meristem. This software accurately annotates all cells within 3D cellular resolution segmentation of dicot SAMs. Cell types identified include the different cell layers representing the L1, L2 and underlying L3 cells, the restricted stem cell niche, and the boundary region between the central zone and organ primordia. Cell types within the primordia are also identified.

## Implementation

The acquisition and 3D cellular segmentation of z-stacks of living plant SAMs have been described previously [3, 11, 16]. The segmentation and polygonal meshing processes are performed within the freely available software MorphoGraphX [11]. 3DCellAtlas Meristem has been implemented within this software to streamline its use and enable widespread distribution and uptake. The code has been implemented in such a way that the users can run 3DCellAtlas Meristem exclusively using the GUI provided within MorphoGraphX.

**Fig. 1:**
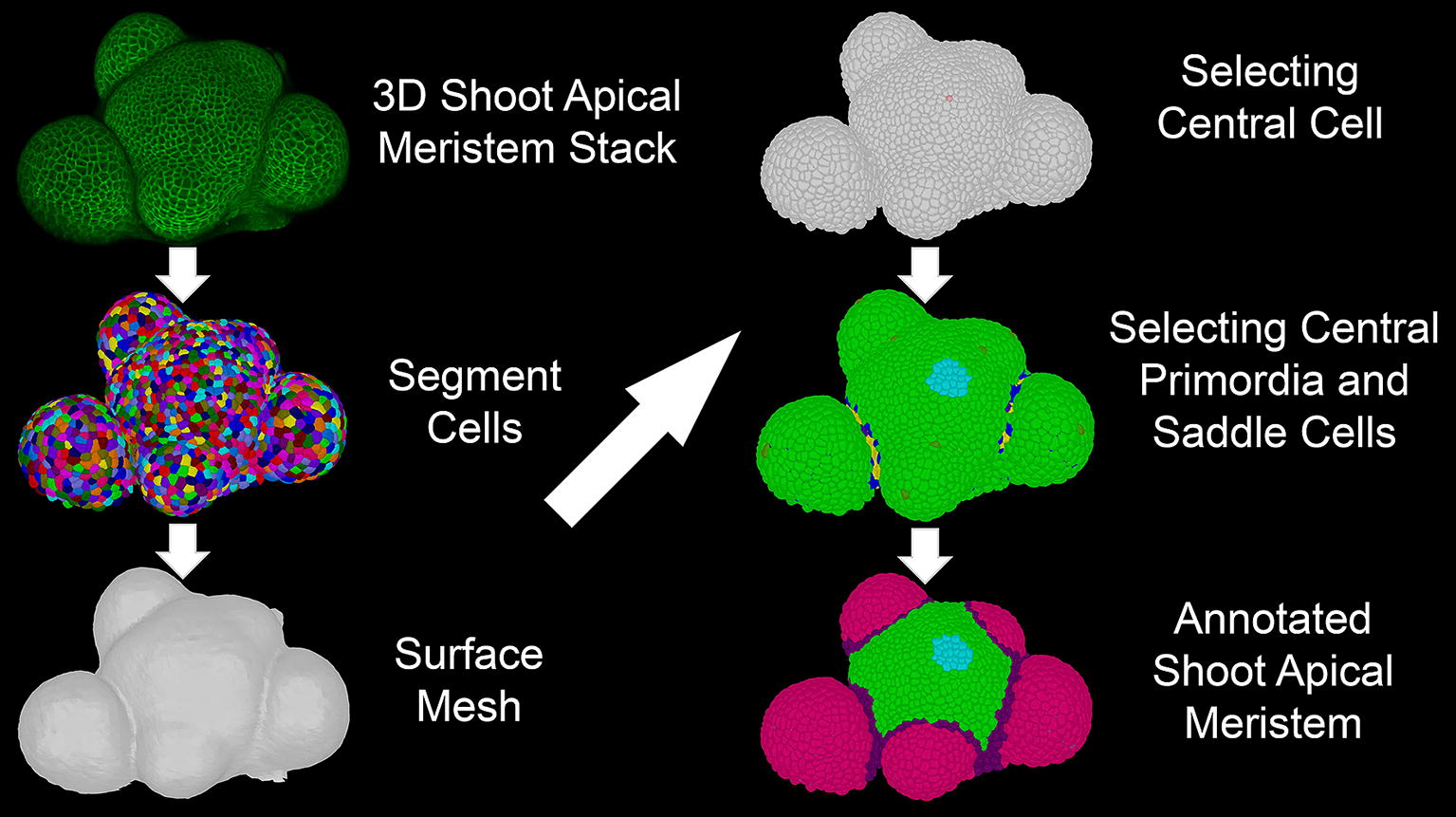
Schematic illustrating the workflow of 3DCellAtlas Meristem

Following the 3D segmentation of the cells in the SAM [11, 16], a second mesh describing the surface of the SAM is generated as described previously [28] (Fig. 1, Additional file 1).

**Fig. 2:**
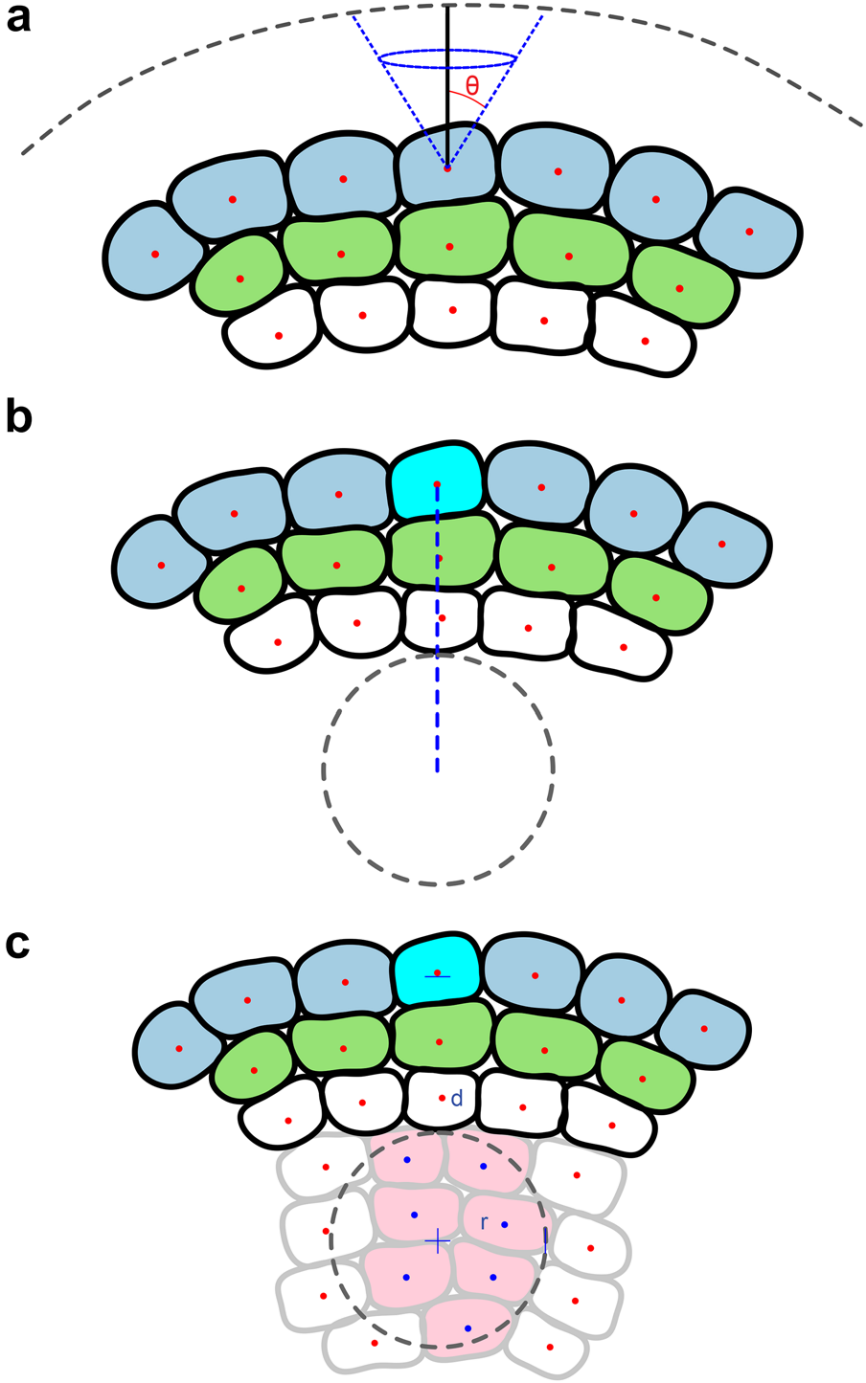
**a** Schematic illustrating the use of cones to define cell axes relative the surface of the SAM. **b** Definition of depth at which the organizing centre is identified indicated as a blue line. **c** The radius of cells comprising the organizing centre show in the grey dashed line, and selected cells in pink. Both the depth and radius used to identify these cells are defined by the user

The first process “Label Meristem” then proceeds to perform the primary annotation of all cells in the SAM. A parameter called “Minimum Cell Volume” enables the user to exclude cells from the analysis which are below a certain cell size. The identification of cell position across the successive layers of the meristem (L1–L3) is then achieved by calculating the centroid ${\mathbf{x}}_{c}^{i}$ of each cell $i$ in the meristem in the manner previously described [11, 28]. For each centroid, the nearest point on the surface mesh ${\mathbf{x}}_{t}^{i}$ is then calculated, forming a vector ${\mathbf{t}}^{i} = {\mathbf{x}}_{c}^{i} - {\mathbf{x}}_{t}^{i}$ for each cell. This vector induces the axis of a cone ${\mathcal{M}}^{i}$ for each cell, with the cell centroid at the vertex, and the nearest point on the surface mesh at the centre of the base (Fig. 2a). Then, for each cell centroid ${\mathbf{x}}_{c}^{j} , j \ne i,$ we check if the centroid lies within the cone ${\mathcal{M}}^{i}$ using the formula

$${\mathbf{x}}_{c}^{j} \in { \mathcal{M}}^{i} {\text{ iff }} \frac{{\left( {{\mathbf{x}}_{c}^{j} - {\mathbf{x}}_{c}^{i} } \right) \cdot {\mathbf{t}}^{i} }}{{\left\| {{\mathbf{x}}_{c}^{j} - {\mathbf{x}}_{c}^{i} } \right\|\left\| {{\mathbf{t}}^{i} } \right\|}} < \cos \theta ,$$

where $\theta$ is the semi-cone angle of the cone ${\mathcal{M}}^{i}$, a variable parameter chosen to be 60°. Thus, the L1 cells are chosen as the cells which have no other centroids inside their cones. The cone angle $\theta$ can be modified to accommodate differences in the sizes of the cells being analysed, for example in different species or in mutant meristems. The L1 cells are then removed from the analysis, and the process is repeated to identify the L2 cells, and then repeated again to identify the L3 cells. All cells below the L2 layer are given the same annotation identity.

The next step named “Mark Meristem” enables the user to define the stem cell niche, or *WUSCHEL* zone [5], within the central region of the meristem. Here the user selects the cell at the top of the dome of the meristem, marking the centre of the region where the stem cell niche resides. By adjusting the parameter for the “Depth of the Organ Centre”, the distance of the stem cell niche from the surface can be altered (Fig. 2b). The Radius parameter adjusts how wide the region selected is (Fig. 2c). This process calls upon “Detect Layers” to mark the L1 and L2, and all cells below the L2 are marked as L3, however the stem cell niche is not overwritten by the L3 label, nor are the cells above it within the L2 layer.

The final stage of the procedure allows for the separate identification and annotation of the primordia within the sample, and the boundary region between these developing organs and the central SAM. Here, users select each primordium individually by clicking a cell on the top of the mass of cells, and a cell in the saddle (boundary) region between the primordium and central SAM. The Boolean feature “Primordium Label Same” can be set to “No”, such that each time a primordium is selected it is given different cellular annotations, separating one primordium from the next. The “Ratio Parameter” defines how large the boundary region is between the primordium and SAM. The “Absolute Distance Parameter” defines how deep the boundary region is. Primordia can be sequentially selected by iteratively running the “Mark Primordium” process.

The centroids of each cell then provide a set of three different coordinates $\varvec{x}_{SAM} , \varvec{x}_{p} , \varvec{ x}_{b}$, which represent the 3D locations of the SAM peak, primordium peak, and boundary saddle respectively. The distances $d_{SAM} = \left\| {\varvec{x}_{SAM} - \varvec{x}_{b} } \right\|$ and $d_{p} = \left\| \varvec{x}_{p} - \varvec{x}_{b} \right\|$ then provide a ratio for a weighted Voronoi map for the cell centroids, such that for all cells $i$ in the sample

$$d_{p}^{i} = \left\|\varvec{x}_{i} - \varvec{x}_{p}\right\| ,\quad d_{SAM}^{i} = \left\|\varvec{x}_{i} - \varvec{x}_{SAM} \right\|,\quad P = \left\{ {i, s.t. \frac{{d_{p}^{i} }}{{d_{SAM}^{i} }} < \frac{{d_{p} }}{{d_{SAM} }}} \right\}.$$

The primordium $P$ is the set of cells with centroids that are relatively closer to the cell at the peak of the primordium than the peak of the SAM, with weighting given by the ratio of the distance from the primordium peak to the boundary, and the distance from the SAM peak to the boundary. This definition may be modified to include cells in the boundary with a small distance $\delta$ such that the Primordium, Boundary, and SAM are the sets $P, B, S$,

$$P = \left\{ {i, s.t. \frac{{d_{p}^{i} }}{{d_{SAM}^{i} }} < \frac{{d_{p} }}{{d_{SAM} }} - \delta } \right\},$$

$$B = \left\{ {i, s.t. \frac{{d_{p} }}{{d_{SAM} }} - \delta \le \frac{{d_{p}^{i} }}{{d_{SAM}^{i} }} \le \frac{{d_{p} }}{{d_{SAM} }} + \delta } \right\},$$

$$S = \left\{ {i, s.t. \frac{{d_{p}^{i} }}{{d_{SAM}^{i} }} > \frac{{d_{p} }}{{d_{SAM} }} + \delta } \right\},$$

giving the final delineation.

## Results

**Fig. 3:**
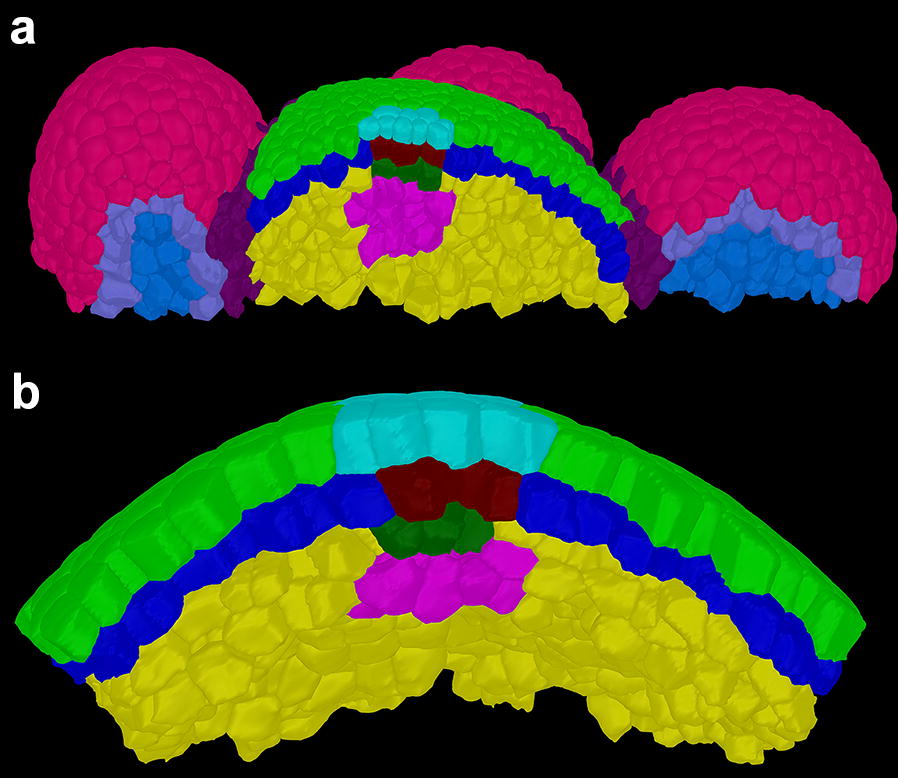
Cellular annotation of SAMs in **a** *Arabidopsis* and **b** tomato. L1 is indicated in light green, L2 in blue, L3 in yellow. Associated layers above the organizing centres are cyan, maroon, and dark green, respectively. The organizing centre is in light pink. The cell layers in the primordia of the *Arabidopsis* meristem (**a**) are given distinct colours

We followed this procedure using *Arabidopsis* floral meristems and tomato vegetative meristems to test the accuracy with which cell types can be identified. The procedure resulted in the comprehensive annotation of all segmented cells within samples (Fig. 3).

To evaluate the effectiveness of this method, we calculated the accuracy by which cells are correctly identified in the SAM (Table 1). We did not include the boundary zone in this analysis as it requires a genetic marker to be properly identified [3].

**Table 1:** Percentage accuracy for the cellular annotation of layers in tomato and *Arabidopsis* SAMs

Cell type	Tomato (%)	*Arabidopsis* (%)
L1	99.8	99.2
L2	99.8	96.5
L3	99.3	96.3

Calculations are based on the percentage of cells that were correctly annotated

The accuracy of this method principally depends upon both the correct 3D segmentation of cells [2, 39], and creation of a surface mesh that fits the SAM properly (see Additional file 1) [11]. The extent to which cells are accurately segmented depends on a number of factors including image acquisition, post-processing, and editing [1, 10]. The degree of user involvement in the correct segmentation of cells will likely diminish over time as adaptive computational approaches to achieve this are developed [14, 25, 32].

In the tomato SAM [11] a very small fraction of cells were not correctly identified, resulting in a greater than 99% accuracy. Cells in the *Arabidopsis* SAM [19] were identified with slightly less accuracy in the lower layers at 96%.

As there is no current method to annotate the cells of the SAM, it was not possible to compare the accuracy of this to other published methods.

**Fig. 4:**
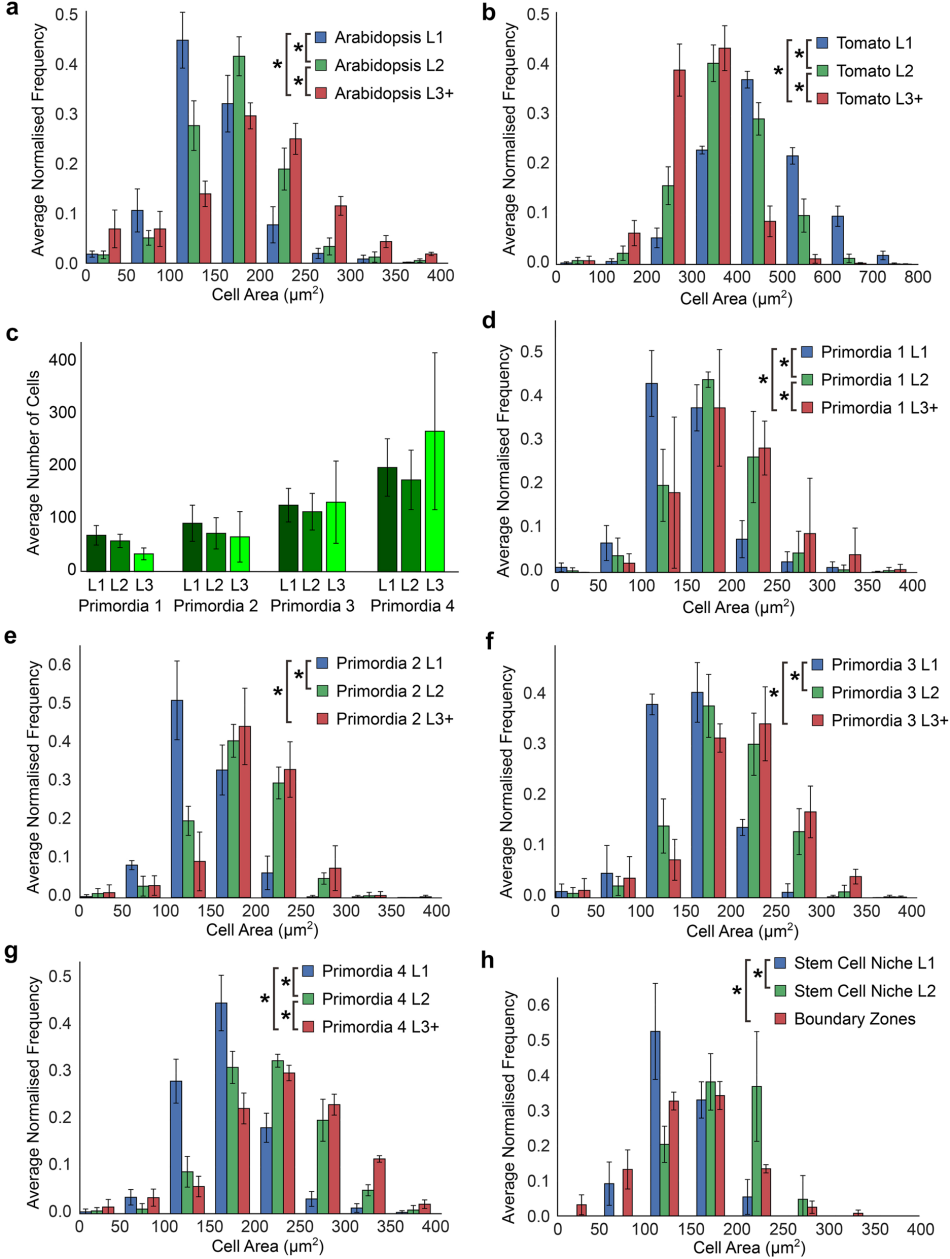
Comparison of size in distinct cell types identified using 3DCellAtlas Meristem. **a** Cell sizes in the L1–L3 in the *Arabidopsis* SAM. **b** Same as **a** with the tomato SAM. **c** Cell number in primordia 1 through 4 in each the L1–L3 in *Arabidopsis*. **d** Cell sizes in the L1–L3 of floral primordia 1 in *Arabidopsis*. **e** Same as **d** with primordia 2. **f** Same as **d** with primordia 3. **g** Same as **d** with primordia 4. **h** Cells sizes in the stem cell niche and boundary zones in the *Arabidopsis* SAM. An asterisk denotes significance at the *p* < 0.05 level (*t* test with Bonferroni corrected *p* value, *p* < 1.08 × 10^−3^)

Having accurately identified cell types in each tomato and *Arabidopsis* SAMs, we quantified the geometric properties of cells across cell layers L1–L3 in each of these species. In *Arabidopsis*, cell size is significantly different across each of the layers, with the surface area progressively increasing with increasing depth into the SAM (Fig. 4a). The tomato SAM has a very different structure, with cells in the L1 being the largest and cell size becoming progressively smaller in successive layers (Fig. 4b). This highlights the presence of distinct cellular organization in the SAM of each of these species.

3DCellAtlas Meristem additionally annotates primordia and the cells within these developing structures. We examined the size of cells across this developmental gradient of organ formation in *Arabidopsis*. As expected, the total number of cells in each layer increased across primordium development (Fig. 4c). Cell size in layers in each of the successive primordia followed a similar pattern, with the L1 having the smallest cells and L3 the largest (Figs. 4d–g). This gradient of cell size is shared between developing primordia and the SAM in *Arabidopsis*.

3DCellAtlas Meristem also identifies the stem cell niche in the central zone of the SAM using an area that is defined by the user (Fig. 2). Coupled with this, the boundary regions between the organ primordia and central region of the SAM are also identified (Additional file 1). We compared cell sizes in each the stem cell niche and boundary zones to the L3 cells of the SAM to identify whether differences are present. Cells in the boundary zone are significantly larger than those in the stem cell niche or the remaining L3 in *Arabidopsis* (Fig. 4h).

**Fig. 5:**
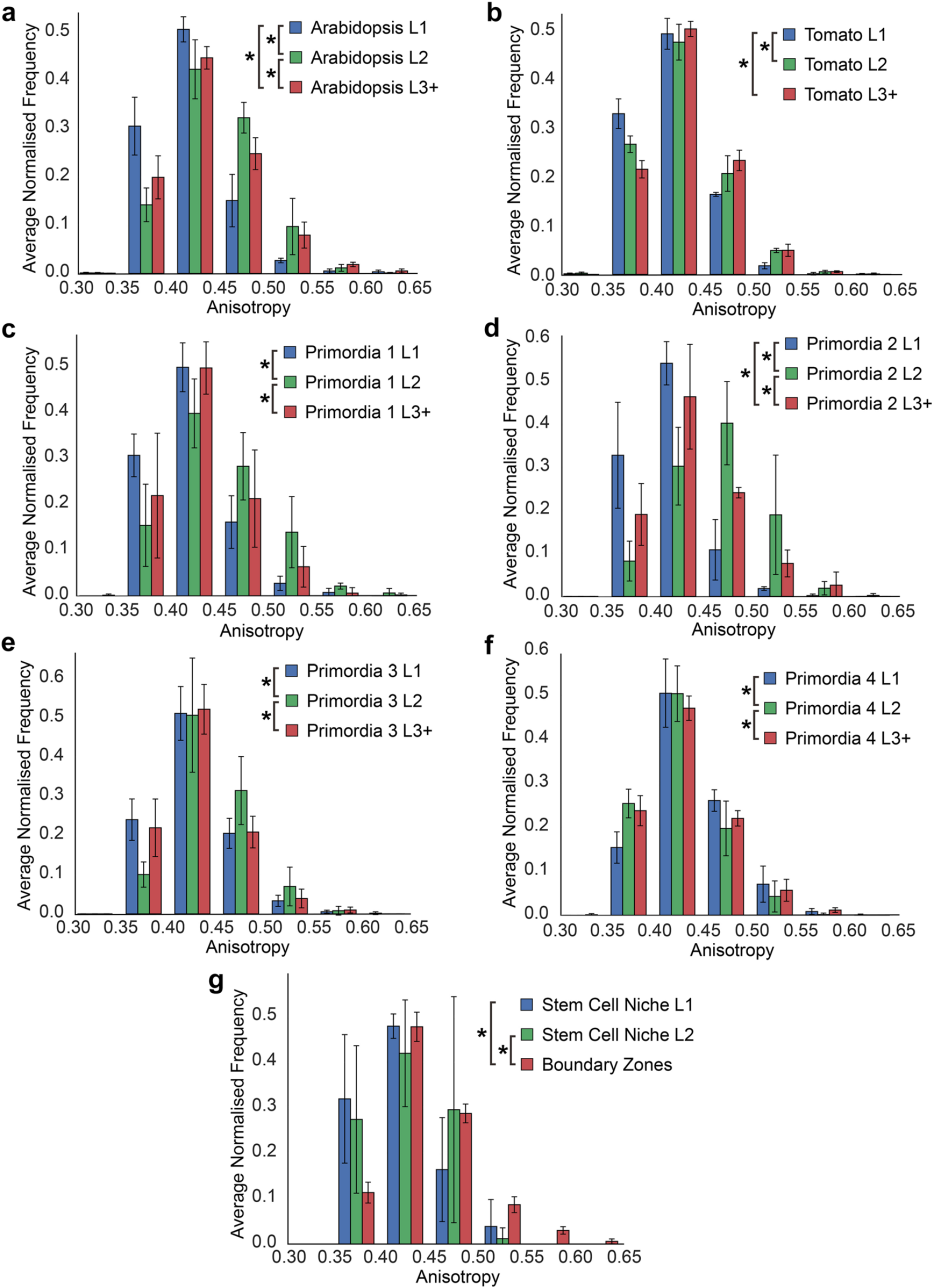
Comparison of cell shape in distinct regions of the SAM identified using 3DCellAtlas Meristem. **a** Cell anisotropy in the L1–L3 in the *Arabidopsis* SAM. **b** Same as **a** with the tomato SAM. Cell anisotropy in the L1–L3 of **c**–**f** floral primordia 1 through 4 in *Arabidopsis*. **g** Cells anisotropy in the stem cell niche and boundary zones in the *Arabidopsis* SAM. An asterisk denotes significance at the *p* < 0.05 level (*t* test with Bonferroni corrected *p* value, *p* < 1.08 × 10^−3^)

Having characterized the distribution of cell sizes across distinct cell populations of the SAM in tomato and *Arabidopsis*, we next sought to examine the distribution of cell shapes based on their anisotropy. Cells in the *Arabidopsis* SAM are most anisotropic in the underlying L3 layer and become progressively more isotropic towards the L1 (Fig. 5a). A similar trend is observed in the tomato SAM (Fig. 5b). This illustrates a conserved gradient of cell shape between these species, in contrast to the divergent distribution of cell sizes (Fig. 4a, b).

Within the developing primordia a similar trend was observed, where the L2 cells were most anisotropic, and the L1 and L3 less so (Fig. 5c–f). A comparison of the boundary zone to the stem cell niche revealed that the stem cells are the most isotropic and boundary zone cells the most anisotropic (Fig. 5g).

**Fig. 6:**
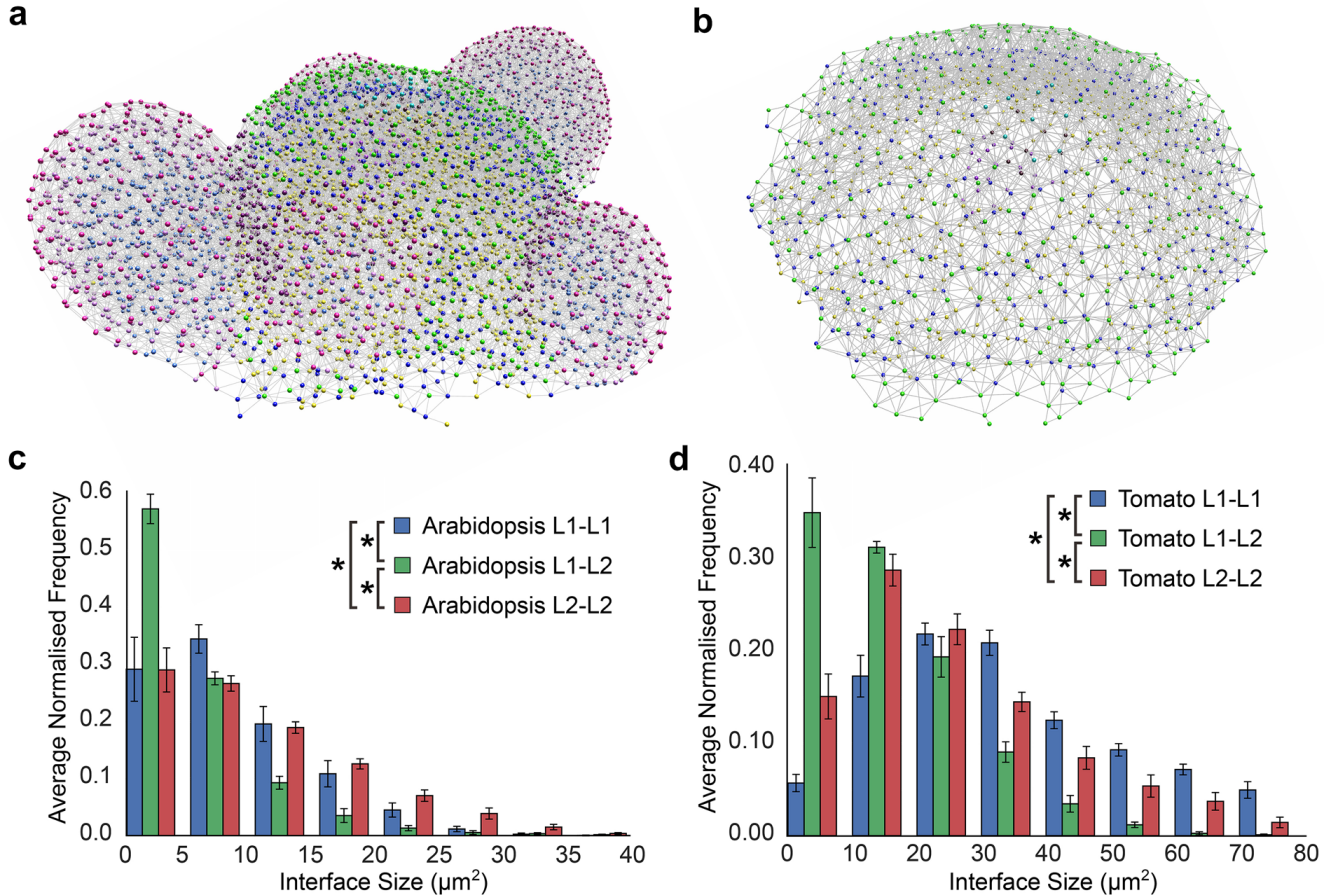
Topology of SAM layers, identified using 3DCellAtlasMeristem. **a** The *Arabidopsis* cellular connectivity network, with node coloured by cell type identified with 3DCellAtlasMeristem. **b** The tomato cellular connectivity network coloured by different cell layers. **c** Cell interface sizes within and between layers of the *Arabidopsis* SAM. **d** Same as **c** with the tomato SAM. An asterisk denotes significance at the *p* < 0.05 level (*t* test with Bonferroni corrected *p* value, *p* < 1.08 × 10^−3^)

The movement of information across the multicellular SAM occurs principally through the shared interfaces between adjacent cells [30, 35]. We sought to understand how the size of shared intercellular interfaces are distributed across each the *Arabidopsis* and tomato SAM based on the cell type annotations derived using 3DCellAtlas Meristem. We made use of our previously published algorithm to identify physical associations between cells in segmented SAMs [28], and in turn represent these as global cellular interaction networks (Fig. 6a, b).

In addition to identifying which cells are in contact with one another, the script is also capable of calculating the size of the shared intercellular interfaces. We plotted the distribution of these intercellular interfaces within each layer and between the L1 than the L2 separately. In both *Arabidopsis* and tomato, the shared interface between the layers is smaller than within the layers (Fig. 6c, d). Interface sizes are greater within the L2 than the L1 in *Arabidopsis* (Fig. 6c), and greater within the L1 and L2 in tomato SAMs (Fig. 6d). This reflects the larger cell sizes in the L1 in tomato and L2 in *Arabidopsis* (Fig. 4a, b). Collectively this reveals a similar cellular architecture to be present within each tomato and *Arabidopsis* SAMs, underpinning the intercellular path of molecular movement through these multicellular systems. In light of the need for information to move across layers in the SAM, for example in the *WUSCHEL*-*CLAVATA1* loop which mediates stem cell homeostasis [36], these genetic programs are acting across similar multicellular templates in different species.

## Materials and methods

### Image acquisition

Images of tomato (*Solanum lycopersicum*) and *Arabidopsis thaliana* meristems were performed using living tissues and an upright Leica SP8. Tomato meristems were stained using propidium iodide as described previously [23]. *Arabidopsis* meristems were imaged using a plasma membrane localized YFP construct described previously [38].

### 3D Cell segmentation

The autoseeded 3D watershed algorithm was used to perform cellular segmentations as described previously [2, 11].

### Cell shape analysis

Anisotropy was calculated using the PCAnalysis process in MorphoGraphX, which abstracts the shape of each cell into three principal vectors. The magnitudes of these vectors are each divided by the sum of all three vector magnitudes, and the maximum resulting value is used to define anisotropy.

### Topological analyses

Extraction of cellular connectivity networks was performed as described previously [20, 28]. Analyses were performed using NetworkX in Python [31].

## Conclusion

The ability to semi-automatically annotate all cells in diverse plant SAMs provides numerous exciting opportunities to analyse the structure of these cellular assemblies. The method described here works for dome-shaped meristems, and serves its function at high accuracy. In addition to the geometric analysis of cell shapes (Figs. 4, 5), this method may be used to understand cell type specific topological properties of the multicellular assemblies within the SAM (Fig. 6). As a proof of concept we were able to identify differences in each of these domains between *Arabidopsis* and tomato SAMs.

The compatability of datasets with this method is facilitated by the inclusion of adaptive controls which allow for the adjustment of key parameters needed to achieve high accuracy annotations. Details of this are included in the User Guide.

The use of fluorescence-based images with 3DCellAtlas enables the simultaneous use of reporter constructs within this context [11]. A boundary marker may be used to delineate cells and perform segmentation, while genetic reporters and biosensors can be integrated in a second channel. MorphoGraphX enables the single cell quantification of reporters and thus paves the way for digital single cell analysis of diverse reporter constructs within the context of the SAM, as has been reported previously for radially symmetric tissues [28].

This approach further enables cell type specific phenotyping of SAMs in plants which carry mutations resulting in both morphological and genetic perturbations. The integration of this software into the popular and freely available software MorphoGraphX [11], where 3D cellular segmentation is routinely being performed, will enable the rapid and seamless adoption of this novel software, adding value to existing and novel datasets.

## Additional file

**Additional file 1.** User guide for 3DCellAtlas Meristem.

## Abbreviations

SAM: shoot apical meristem
L1, L2, L3: layer 1, 2, 3

## References

[1] Bassel GW (2015). Accuracy in quantitative 3D image analysis. Plant Cell.

[2] Bassel GW, Stamm P, Mosca G, de Reuille PB, Gibbs DJ, Winter R, Janka A, Holdsworth MJ, Smith RS (2014). Mechanical constraints imposed by 3D cellular geometry and arrangement modulate growth patterns in the *Arabidopsis* embryo. Proc Natl Acad Sci USA.

[3] Besnard F, Refahi Y, Morin V, Marteaux B, Brunoud G, Chambrier P, Rozier F, Mirabet V, Legrand J, Lainé S (2014). Cytokinin signalling inhibitory fields provide robustness to phyllotaxis. Nature.

[4] Boudon F, Chopard J, Ali O, Gilles B, Hamant O, Boudaoud A, Traas J, Godin C (2015). A computational framework for 3D mechanical modeling of plant morphogenesis with cellular resolution. PLoS Comput Biol.

[5] Brand U, Fletcher JC, Hobe M, Meyerowitz EM, Simon R (2000). Dependence of stem cell fate in *Arabidopsis* on a feedback loop regulated by CLV3 activity. Science.

[6] Breuer D, Nowak J, Ivakov A, Somssich M, Persson S, Nikoloski Z (2017). System-wide organization of actin cytoskeleton determines organelle transport in hypocotyl plant cells. Proc Natl Acad Sci.

[7] Chen F, Tillberg PW, Boyden ES (2015). Expansion microscopy. Science.

[8] Chung K, Wallace J, Kim S-Y, Kalyanasundaram S, Andalman AS, Davidson TJ, Mirzabekov JJ, Zalocusky KA, Mattis J, Denisin AK (2013). Structural and molecular interrogation of intact biological systems. Nature.

[9] Conn A, Pedmale UV, Chory J, Navlakha S (2017). High-resolution laser scanning reveals plant architectures that reflect universal network design principles. Cell Syst.

[10] Cunha AL, Roeder AHK, Meyerowitz EM. Segmenting the sepal and shoot apical meristem of *Arabidopsis thaliana*. In: Annual International Conference of the IEEE Engineering in Medicine and Biology, Buenos Aires, 2010, pp 5338–5342. 2010. 10.1109/IEMBS.2010.5626342 10.1109/IEMBS.2010.5626342 21096072

[11] de Reuille PB, Routier-Kierzkowska A-L, Kierzkowski D, Bassel GW, Schüpbach T, Tauriello G, Bajpai N, Strauss S, Weber A, Kiss A (2015). MorphoGraphX: a platform for quantifying morphogenesis in 4D. Elife.

[12] Dhondt S, Wuyts N, Inzé D (2013). Cell to whole-plant phenotyping: the best is yet to come. Trends Plant Sci.

[13] Duran-Nebreda S, Bassel GW (2017). Bridging scales in plant biology using network science. Trends Plant Sci..

[14] Eschweiler D, Spina TV, Choudhury RC, Meyerowitz E, Cunha A, Stegmaier J. CNN-based preprocessing to optimize watershed-based cell segmentation in 3D confocal microscopy images. 2018. arXiv preprint arXiv:181006933.

[15] Fahlgren N, Gehan MA, Baxter I (2015). Lights, camera, action: high-throughput plant phenotyping is ready for a close-up. Curr Opin Plant Biol.

[16] Fernandez R, Das P, Mirabet V, Moscardi E, Traas J, Verdeil JL, Malandain G, Godin C (2010). Imaging plant growth in 4D: robust tissue reconstruction and line aging at cell resolution. Nat Methods.

[17] Gooh K, Ueda M, Aruga K, Park J, Arata H, Higashiyama T, Kurihara D (2015). Live-cell imaging and optical manipulation of *Arabidopsis* early embryogenesis. Dev Cell.

[18] Hamant O, Heisler MG, Jonsson H, Krupinski P, Uyttewaal M, Bokov P, Corson F, Sahlin P, Boudaoud A, Meyerowitz EM (2008). Developmental patterning by mechanical signals in *Arabidopsis*. Science.

[19] Jackson MDB, Duran-Nebreda S, Kierzkowski D, Strauss S, Xu H, Landrein B, Hamant O, Smith RS, Johnston IG, Bassel GW (2019). Global topological order emerges through local mechanical control of cell divisions in the Arabidopsis shoot apical meristem. Cell Syst..

[20] Jackson MD, Xu H, Duran-Nebreda S, Stamm P, Bassel GW (2017). Topological analysis of multicellular complexity in the plant hypocotyl. Elife.

[21] Jones AR, Forero-Vargas M, Withers SP, Smith RS, Traas J, Dewitte W, Murray JA (2017). Cell-size dependent progression of the cell cycle creates homeostasis and flexibility of plant cell size. Nat Commun.

[22] Keller PJ, Schmidt AD, Wittbrodt J, Stelzer EH (2008). Reconstruction of zebrafish early embryonic development by scanned light sheet microscopy. Science.

[23] Kierzkowski D, Nakayama N, Routier-Kierzkowska AL, Weber A, Bayer E, Schorderet M, Reinhardt D, Kuhlemeier C, Smith RS (2012). Elastic domains regulate growth and organogenesis in the plant shoot apical meristem. Science.

[24] Kuchen EE, Fox S, de Reuille PB, Kennaway R, Bensmihen S, Avondo J, Calder GM, Southam P, Robinson S, Bangham A (2012). Generation of leaf shape through early patterns of growth and tissue polarity. Science.

[25] Liu M, Chakraborty A, Singh D, Yadav RK, Meenakshisundaram G, Reddy GV, Roy-Chowdhury A (2011). Adaptive cell segmentation and tracking for volumetric confocal microscopy images of a developing plant meristem. Mol Plant.

[26] Long F, Peng H, Liu X, Kim SK, Myers E (2009). A 3D digital atlas of *C. elegans* and its application to single-cell analyses. Nat Methods.

[27] Michelin G, Refahi Y, Wightman R, Jönsson H, Traas J, Godin C, Malandain G. Spatio-temporal registration of 3D microscopy image sequences of *Arabidopsis* floral meristems. In: Paper presented at: ISBI-international symposium on biomedical imaging. 2016.

[28] Montenegro-Johnson TD, Stamm P, Strauss S, Topham AT, Tsagris M, Wood AT, Smith RS, Bassel GW (2015). Digital single-cell analysis of plant organ development using 3DCellAtlas. Plant Cell.

[29] Reddy GV, Heisler MG, Ehrhardt DW, Meyerowitz EM (2004). Real-time lineage analysis reveals oriented cell divisions associated with morphogenesis at the shoot apex of *Arabidopsis thaliana*. Development.

[30] Rinne PL, Welling A, Vahala J, Ripel L, Ruonala R, Kangasjarvi J, van der Schoot C (2011). Chilling of dormant buds hyperinduces FLOWERING LOCUS T and recruits GA-inducible 1,3-beta-glucanases to reopen signal conduits and release dormancy in Populus. Plant Cell.

[31] Schult DA, Swart P. Exploring network structure, dynamics, and function using NetworkX. In: Paper presented at: proceedings of the 7th Python in science conferences (SciPy 2008). 2008.

[32] Spina TV, Stegmaier J, Falcão AX, Meyerowitz E, Cunha A. SEGMENT3D: A web-based application for collaborative segmentation of 3D images used in the shoot apical meristem. In: Paper presented at: 2018 IEEE 15th international symposium on (IEEE) biomedical imaging (ISBI 2018). 2018.

[33] Susaki EA, Tainaka K, Perrin D, Kishino F, Tawara T, Watanabe TM, Yokoyama C, Onoe H, Eguchi M, Yamaguchi S (2014). Whole-brain imaging with single-cell resolution using chemical cocktails and computational analysis. Cell.

[34] Truernit E, Bauby H, Dubreucq B, Grandjean O, Runions J, Barthelemy J, Palauqui JC (2008). High-resolution whole-mount imaging of three-dimensional tissue organization and gene expression enables the study of Phloem development and structure in *Arabidopsis*. Plant Cell.

[35] Tylewicz S, Petterle A, Marttila S, Miskolczi P, Azeez A, Singh R, Immanen J, Mähler N, Hvidsten T, Eklund D (2018). Photoperiodic control of seasonal growth is mediated by ABA acting on cell–cell communication. Science.

[36] Weigel D, Jurgens G (2002). Stem cells that make stems. Nature.

[37] Willis L, Refahi Y, Wightman R, Landrein B, Teles J, Huang KC, Meyerowitz EM, Jönsson H (2016). Cell size and growth regulation in the *Arabidopsis thaliana* apical stem cell niche. Proc Natl Acad Sci.

[38] Yang W, Schuster C, Beahan CT, Charoensawan V, Peaucelle A, Bacic A, Doblin MS, Wightman R, Meyerowitz EM (2016). Regulation of meristem morphogenesis by cell wall synthases in *Arabidopsis*. Curr Biol.

[39] Yoshida S, de Reuille PB, Lane B, Bassel GW, Prusinkiewicz P, Smith RS, Weijers D (2014). Genetic control of plant development by overriding a geometric division rule. Dev Cell.

