# Supplementary material for: 3DCellAtlas Meristem: a tool for the global cellular annotation of shoot apical meristems
Source: Plant Methods. 2019 Apr 4;15:33. doi: 10.1186/s13007-019-0413-0 (PMC6448224; doi:10.1186/s13007-019-0413-0)
Supplement: Supplementary file 1 — Additional file 1. User guide for 3DCellAtlas Meristem. [file 13007_2019_413_MOESM1_ESM.pdf]

3DCellAtlas Meristem User Guide

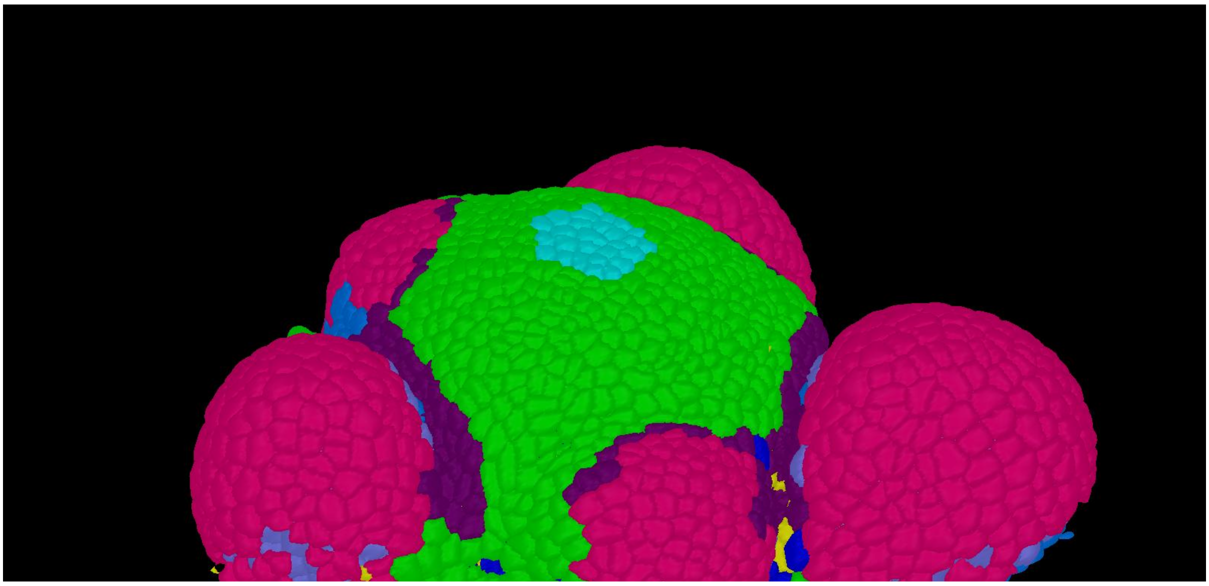

INTRODUCTION

Cell Atlas 3D Meristem is a tool that can be used to label cells in a 3D shoot apical meristem image with minimal user input.

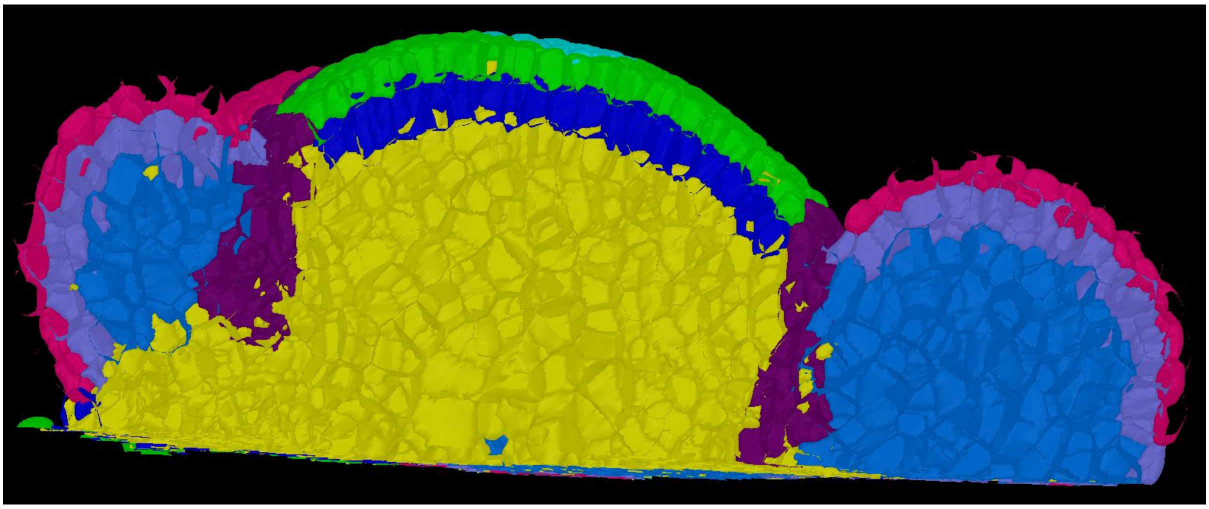

Figure 1. *Arabidopsis* SAM with cell types identified using 3DCellAtlas Meristem and given distinct colours for visualization.

BEFORE GETTING STARTED

The effectiveness of this tool depends on having an accurately segmented SAM in the first instance. If cells are not segmented properly, their analysis will yield inaccurate data while this tool will also be challenged to identify cell types. It is very important that the quality of the 3D cell segmentation is high because any mistakes during segmentation will carry forward into the cell mesh and these may be amplified in the final output.

The segmentation need only be perfectly resolved to at least 3 cell layers of depth for best performance. As the resolution of the fluorescent signal from confocal imaging of the SAM decreases deeper into the meristem, it may not be possible to resolve cells at this depth. A good image with strong and clearly resolved boundary marker signal deep into the sample will facilitate this.

Once you have constructed a mesh representing 3D cell surfaces, a process can be followed to identify segmentation errors. Cells which are merged together (under segmented) cannot be separated, and a lower watershed segmentation threshold will need to be used. It is recommended to save work regularly when segmenting and to err on the side of caution when merging cells together as this is irreversible.

PROTOCOL STEPS:

Step 1. Segmenting cells in 3D. Protocols describing this process have been published previously (REFS). Briefly, apply a Gaussian Blur at a radius of 0.3 in all directions and perform an autoseeded watershed segmentation. Following segmentation, if there is a single very large ‘cell’ at the bottom of your mesh (an artefact of the loss of image quality this deep into meristem) it can be deleted by

selecting it with Select Connected Area tool in the left hand column, selecting the cell with alt + left click and pressing delete.

Marching cubes 3D may be used to generate polygonal meshes describing cell surfaces. Set Cube Size to 1 µm and Smooth Passes to 6, resulting in an accurate segmentation. Decreasing the cube size further or increase the smooth passes is unnecessary and the resulting mesh will take a very long time to initialize each time it is loaded in. Do not adjust the other settings (Min Voxels and Label). The output of this process is shown in Figure 2.

Once your mesh has finished rendering it can be saved using “Save” from the Mesh 1 menu at top of the MGX window.

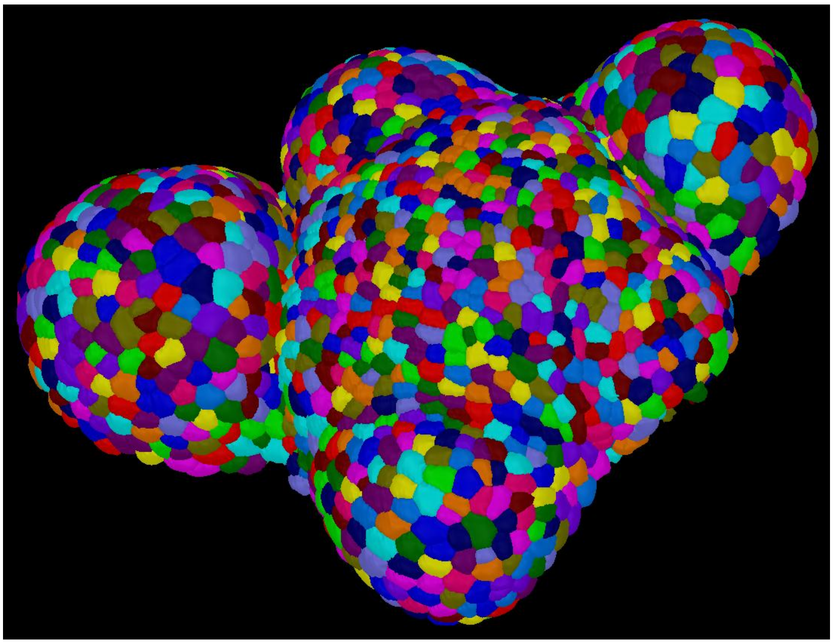

Figure 2. Cells in the *Arabidopsis* SAM segmented in 3D and meshed using Marching Cubes 3D. Distinct cells are given unique colours for visualization.

Step 2. Generating a surface mesh.

A surface mesh describes the gross morphological geometry of the cellular structure. 3DCellAtlas Meristem makes use of a surface mesh to orient the process which identifies successive layers of cells in the SAM.

In order to achieve good results with 3DCellAtlas Meristem, it is crucial that a high quality surface mesh is used. In order to compute which cells should belong to the first layer, the program calculates the distances from cell centroids to the surface mesh. Therefore, a poorly fitting surface mesh will lead to errors in which cells are designated as layer 1 and this will in turn lead to further errors in other layers.

In order to construct a surface mesh, the original confocal microscope image must be loaded into the active stack. This should be copied into the work stack and then blurred. A sufficiently high Gausian blur (i.e. 3 in all directions) can be used to distort

all the information in the image except the basic structure. Then create a surface mesh.
Copy Main to Work Stack: Process > Stack > MultiStack > Copy Main to Work Stack
Blur: Process > Stack > Filters > Gaussian Blue Stack
Surface mesh: Process > Mesh > Creation > Marching Cubes Surface
The default cube size of 5 is ok but the threshold you use when create a mesh will depend on the intensity of original confocal image used for blurring. Once the mesh is made, it is likely to be too loosely fitting so will need to be shrunk a little bit. This can be done using the Shrink Mesh tool (in 1 µm increments) but ensure you do overshrink and get overlap. If Shrink Mesh is used, smoothing out the resulting spikes in the mesh surface with the Smooth Mesh tool will be required (Setting the Passes parameter to 10 is recommended to speed this up).
Shrink mesh: Process > Mesh > Structure > Shrink Mesh
Smooth mesh: Process > Mesh > Structure > Smooth Mesh

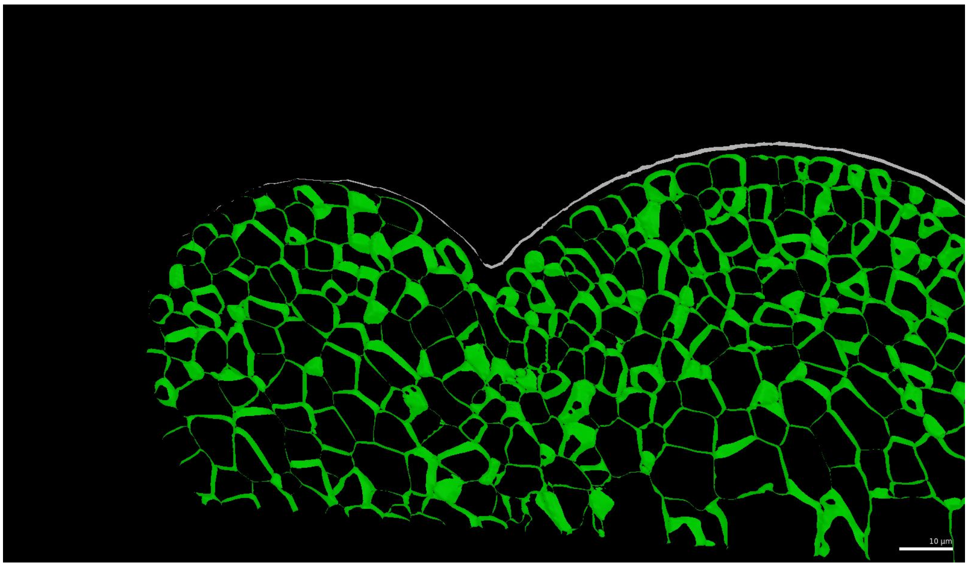

Figure 3. Fitting of the surface mesh to the SAM. A uniform and closely fitting surface mesh which does not touch the cells of the SAM is favoured.

Figure 3 shows what a well fitted mesh should look like. This should hug the top of the meristem as closely and uniformly as possible, although in practice this can be difficult to achieve around primordia, especially in boundary regions. Issues with surface meshes are illustrated below.

Finally, once a surface mesh had been made which accurately reflects the contours of the cell mesh, the bottom should be clipped off the surface mesh (Figure 4). This is to ensure that cells at the bottom of the meristem structure aren't erroneously labelled at belonging to layer 1 when they in fact lie on the edge of the field of view of the microscope that took the original image.
Clipping the bottom of the mesh is best performed using Clip 3 and aligning the grid over the area of the mesh to be clipped. Then highlight the area using Select Clip Region and press delete. Don't forget to save the clipped mesh.
Select Clip Region: Process > Mesh > Selection > Select Clip Region

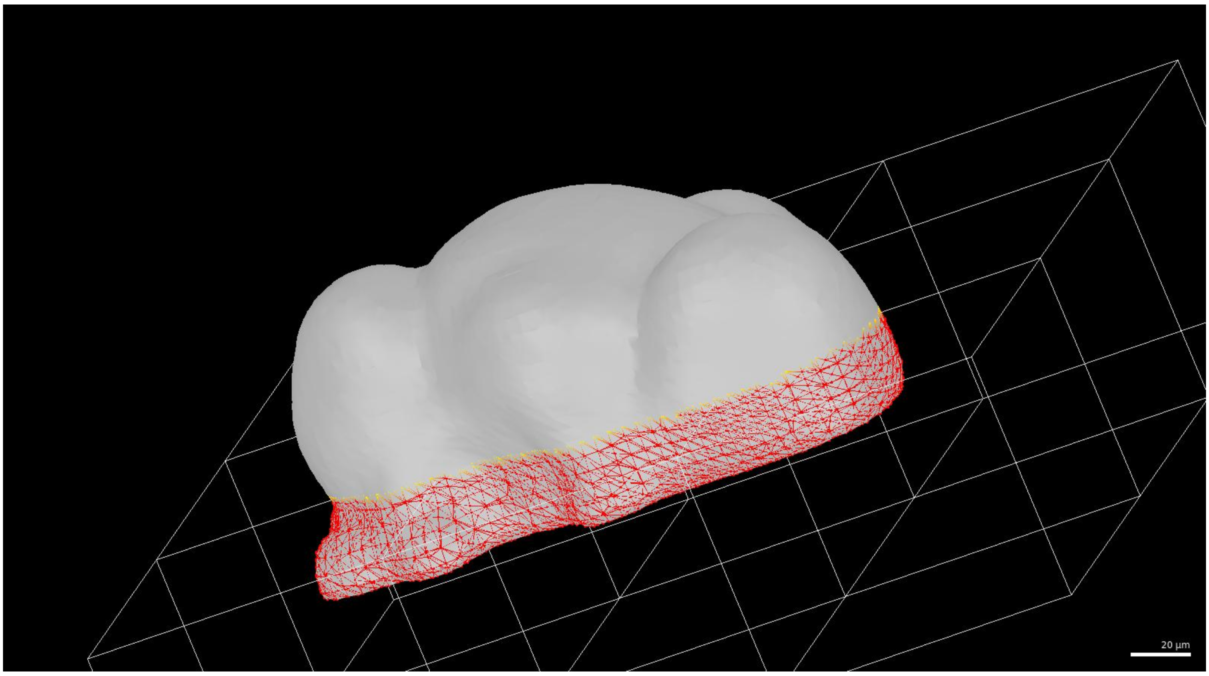

Figure 4. Trimming of the surface mesh.

Poorly fitted surface mesh

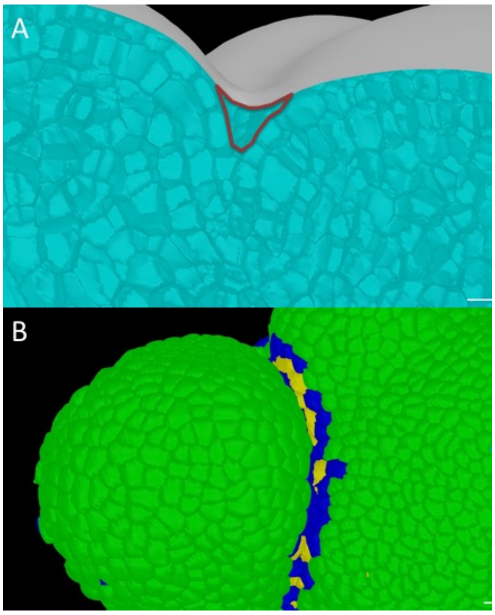

Figure 5. Poor fitting of the surface mesh to the SAM indicated in red in (A). This results in mis-annotation of cells in this saddle region (B).

For instance, in Figure 4, although the surface mesh maps very closely to the cell mesh around the main meristem and the primordia, it does not hug the boundary region as it is too shallow. There is, therefore, a large volume of empty space between the surface mesh and the cells towards the bottom of the boundary region (Figure 5A).

When cones are drawn from these cells to the mesh, the distance from the surface mesh means they are interpreted as belonging to layer 2 and 3 (Figure 5B).

Mesh too close to the sample

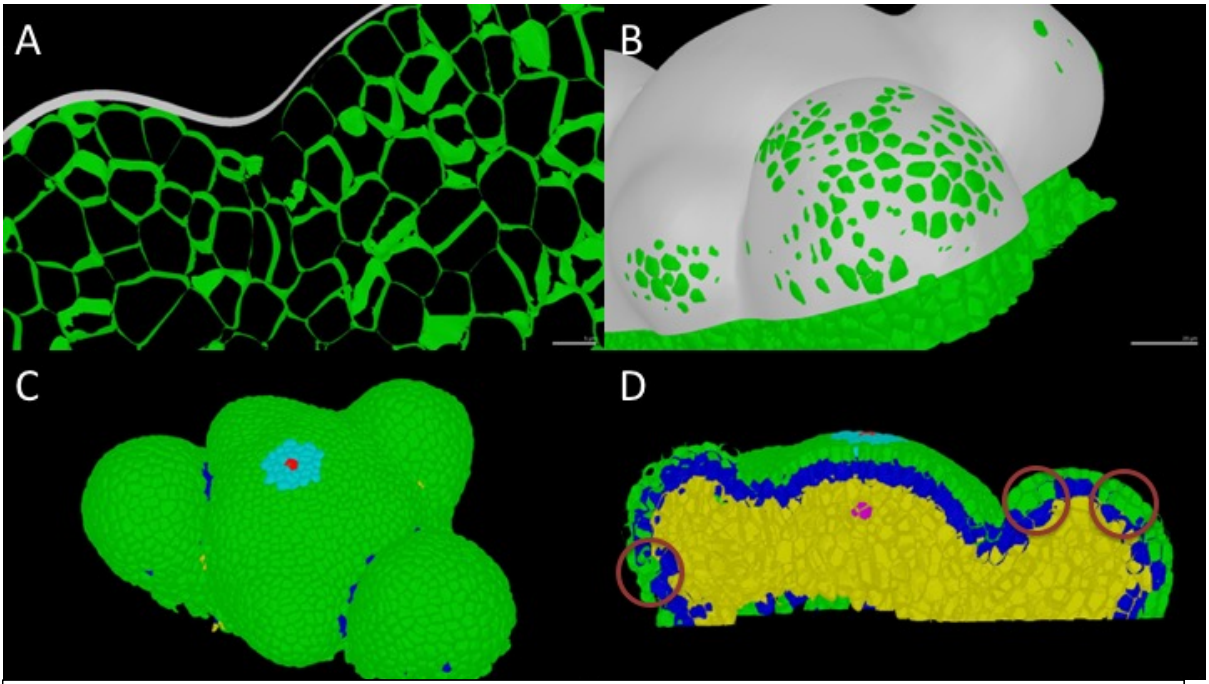

Figure 6. Consequences of the mesh overlapping with cells in the sample (A) - (B) View of an over shrunken mesh. (C) L1 cells appear to be properly annotated however lower layers are mis-annotated (D).

Over-shrinking the surface mesh can closely map the boundary regions. Figure 5 illustrates the consequences of this. When the surface mesh into overlaps with the cell mesh (Figures 6A-B). Very small amounts of overlap can be tolerated by the tool but not to this extent. While the annotation looks very good for the outside layer (Figure 6C), this has caused the tool to identify some cells as layer 1 (Figure 6D) which should actually belong to layer 2.

Step 3. Performing cell annotations.

Once you have a cell mesh and a surface mesh ready, you can now use the tool to identify which layer a cell belongs to. Load the cell mesh in the mesh slot of stack 1 and the surface mesh in the mesh slot of stack 2.
The tool is divided into two parts which will be discussed in more detail later. Firstly, the Label Meristem tool is used to label all cells in the meristem as layers L1 to L3 and also marks cells of the organizing center of the meristem. The second part, Mark Primordia, is used to distinguish cells belonging to a primordium from the rest of the meristem and will overwrite the existing labels of these cells. The two tools must be used in this order.

Label meristem
The tool has been streamlined to allow much of the labelling to occur in a few clicks. The top cell of the main meristem must be selected using the Select Connected Area tool from the left hand column. This cell can be easily found by navigating through clips 1 and 2 and selecting the cell which appears at the apex of both clips. Once this cell is highlighted, the tool can be used.
Label meristem: Process > Mesh > Cell Atlas 3D > Meristem > Label Meristem
There are 4 parameters that can be adjusted for this tool and these will be discussed in the next slides; Minimum Volume, Depth Organizing Center, Radius and Cone Angle. For best results, the following settings are recommended respectively; 10, 0.75, 1.0 and 0.9.

Radius

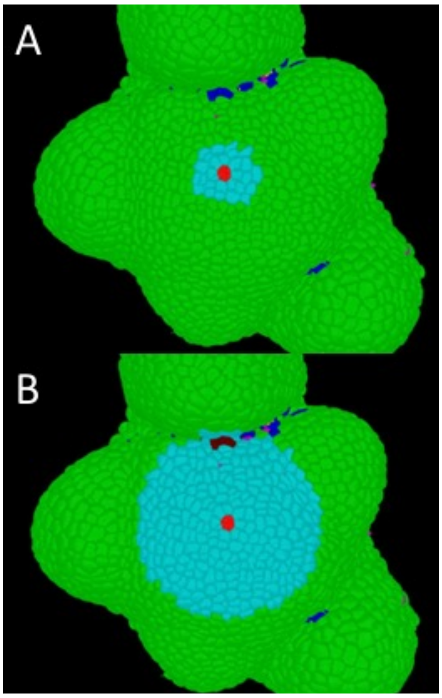

Figure 7. Impact of Radius size setting equal to (A) 1 and (B) 3.

Adjusting the 'Radius' input on the Label Meristem tool will define how far the region labelled as the central zone will extend across the meristem. The default setting is set to 1 (Figure 7A) is appropriate whilst increasing this value to 3 (Figure 7B) results in a much larger area to be set as parents of the organizing centre which will likely interfere with labelling the primordia later.

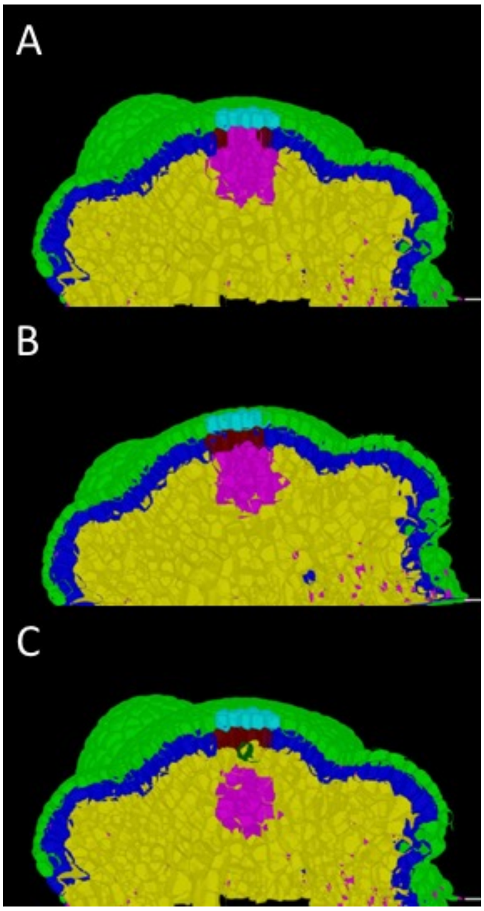

Figure 8. Impact of Depth of Organizing Centre setting equal to (A) 0.4, (B) 0.75 and (C) 1.5.

The ‘Depth Organizing Center’ setting defines how far underneath the cell designated as the top of the central zone should cells be labelled as belonging to the *WUSCHEL* zone (the region labelled in pink). This should be positioned just under the second layer of cells.
If the depth is set too low (Figure 8A, set to 0.4) then the *WUSCHEL* zone protrudes into the top 2 layers. At an intermediate setting (Figure 8B, set to 0.75) the region is correctly positioned whilst if using too high a value (Figure 8C, set to 1.5) it is positioned too deep within the meristem.

Cone Angle
This process drives the identification of successive cell layers.

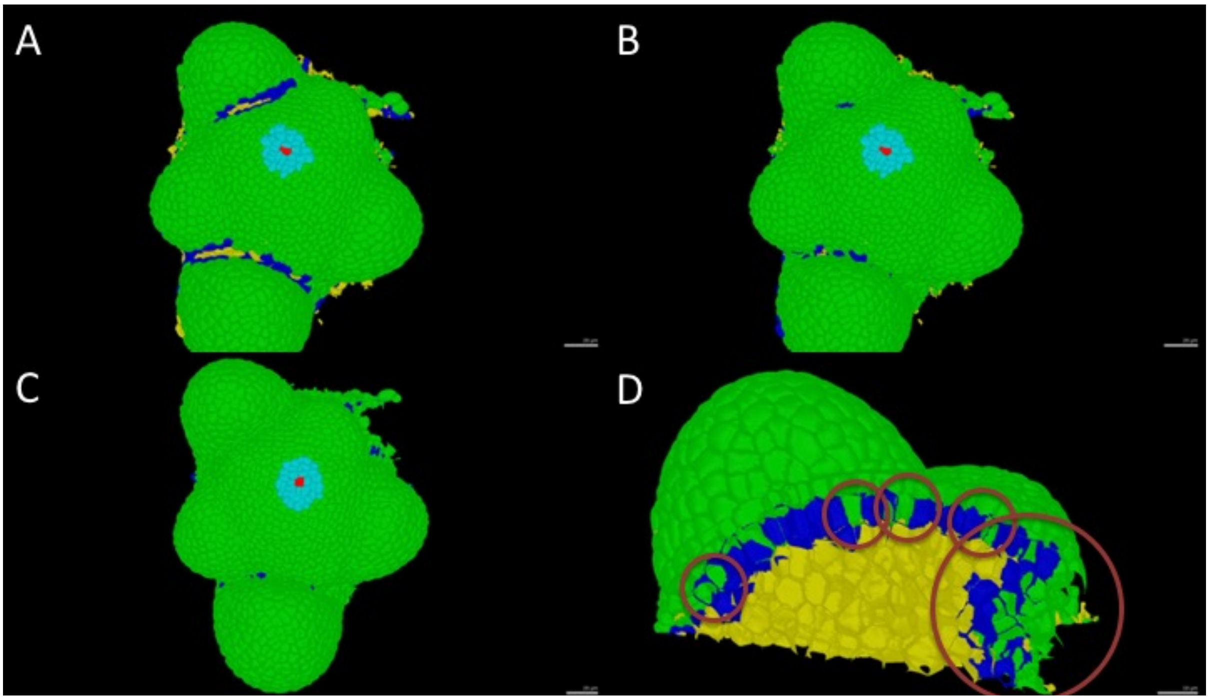

Figure 9. Setting of the cone angle and its consequences. Cone angle set at (A) 0.9, (B) 0.6, (C) 0.4. (D) Mis-annotation of lower layers following the use of a cone angle which is too low.

If the cone angle is set high (Figure 9A, set to 0.9) then many parents in the first layer are incorrectly set as deeper layers but not to an extent that can't be corrected when labelling primordia. At an intermediate or low cone angle (Figure 9B, set at 0.6, Figure 9C, set at 0.4) then the surface results appear to be excellent but inaccuracies are found in lower layers (D).

Step 4. Saving and loading parent labels.

Before starting to label the primordia it is a good idea to save your work so far. If you are not happy with how a primordium has been labelled you can then load up your previous work from before rather then starting again. It is recommended to save a new version of your parent labels each time you satisfactorily resolve a primordium.
Saving parents: Process > Mesh > Lineage Tracking > Save Parents.
Loading parents: Process > Mesh > Lineage Tracking > Load Parents.
If you load in a previous set of parents but they do not visualize on your mesh (for instance, after restarting MGX) this is because the Parents tickbox is unticked. This can be found on the under the Surface section of the stack containing your mesh. If this box is ticked and the parents still aren't displaying, ensure the Surface section is set to display Cells and that the drop down box to the right of this is displaying Labels.

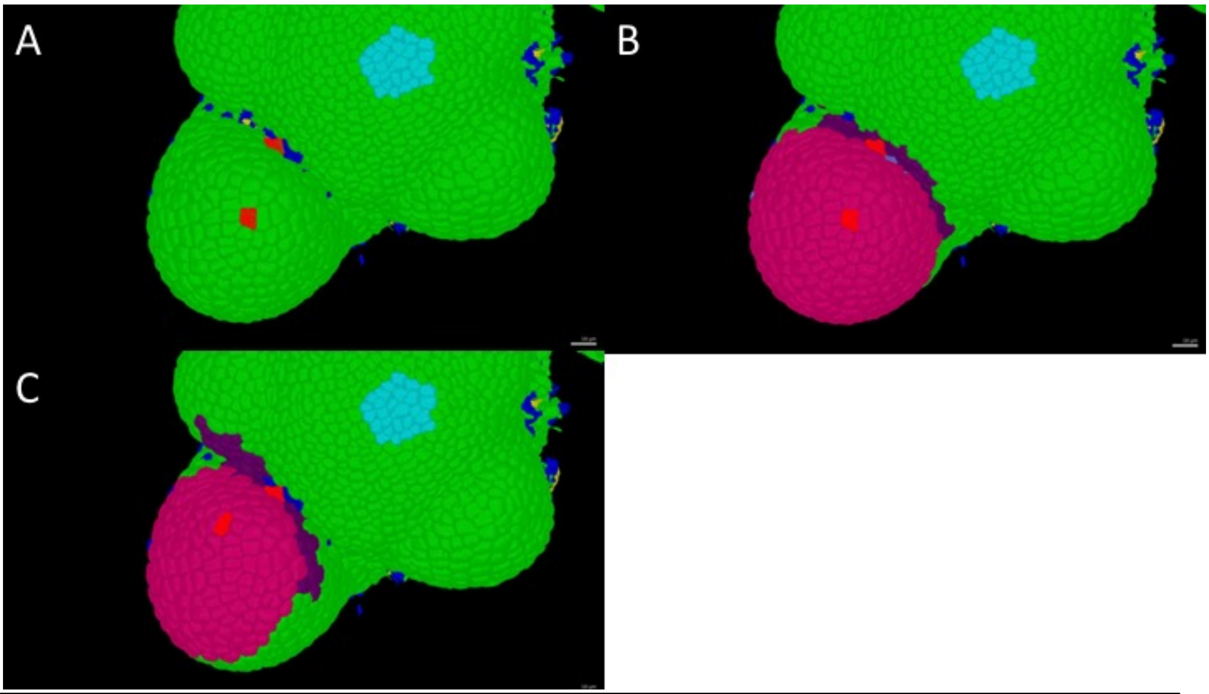

Figure 10. Mark primordia process. (A)-(B) Selection of the top of the primordium and boundary region followed by annotation of cells. (C) Incorrect selection of the top of the primordium and annotation consequences.

In order to designate a primordium, use the Select Connected Area tool from the left hand column (make sure you are in the same stack as your cell mesh) and select the primordial center (the cell at the top of the primordia) and the saddle cell (the cell in the middle of the floor of the boundary region). Once both cells have been selected (Figure 10A) then run Mark Primordium and the primordia will be labelled (Figure 10B). Notice how some incorrectly labelled cells on the floor of the boundary region are overwritten. An incorrect choice of primordial center (Figure 10C) will cause the labelling to shift slightly to one side.
Usually, the default settings for this tool (Ratio Parameter = 0.8, Abs Dis Parameter = 1.3) will give the best results but for some primordia which are tricky to label, these may need modifying. This is covered in more detail in subsequent slides.

Ratio parameter

Adjusting the Ratio Parameter will alter the size of the area of cells designated as boundary region. This input is very sensitive and small changes to the value will affect the size of boundary regions dramatically.

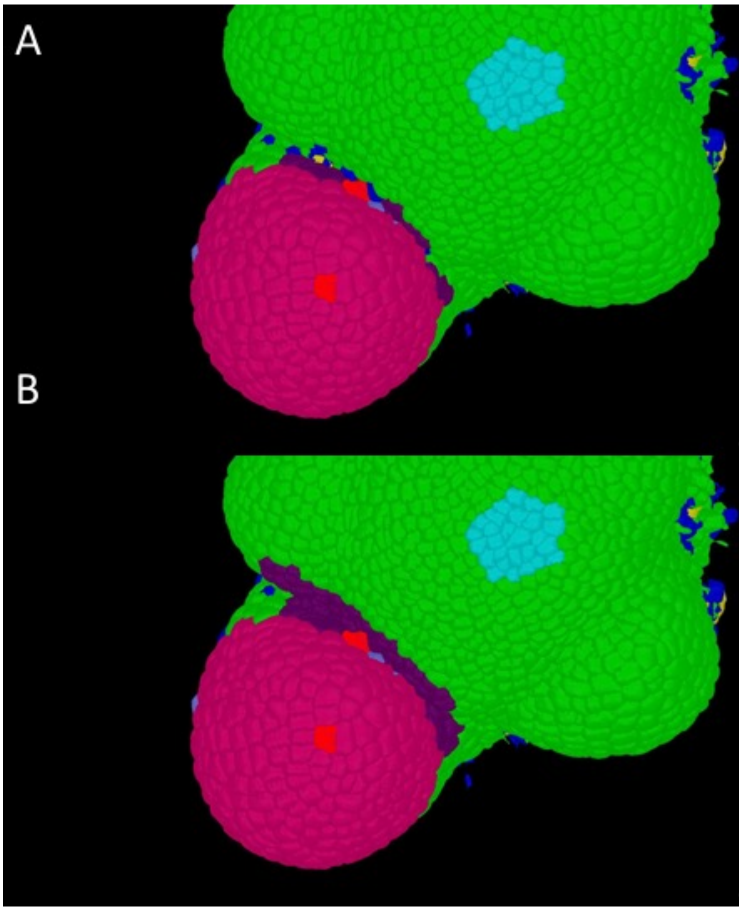

Figure 11. Impact of Ratio Parameter (A) 0.75 and (B) 0.85 on the extent to which the boundary region is labelled.

Increasing the Ratio Parameter (Figure 11A, set to 0.75) will decrease the size of the boundary region whilst lowering the value will actually expand the boundary region (Figure 11B, set to 0.85). In cases where a primordia is proving very difficult to resolve, it may be necessary to adjust this setting but the default value of 0.8 is usually sufficient.

Abs Dis Parameter

This setting influences how deep into the structure the boundary region vertically extends. When the value is low (Figure 12A, set to 1) then the boundary region extends only a couple of layers into the meristem. When an intermediate value is use (Figure 12B, set to 1.3) then the boundary region transcends multiple cell layers. Using a high value (Figure 12C, set to 1.7) results in the boundary region extending to bottom of the structure.

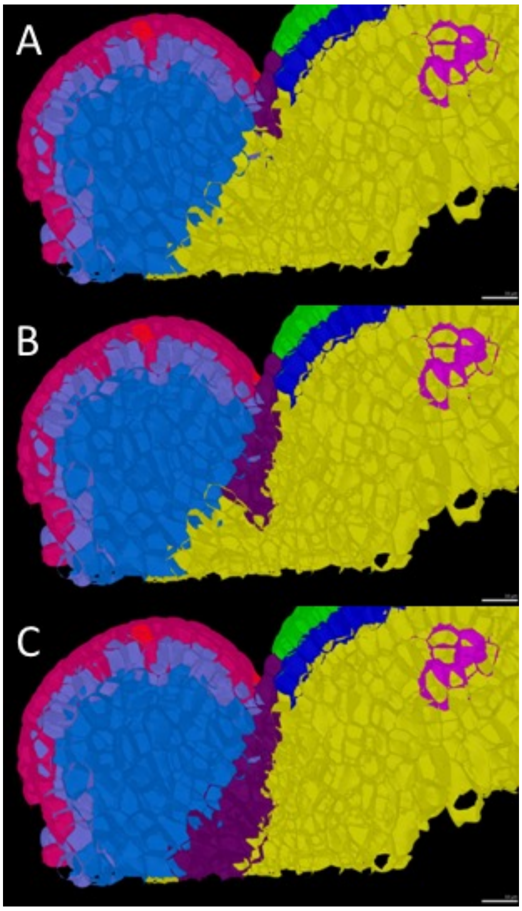

Figure 12. Impact of Abs Dis Parameter (A) 1, (B) 1.3 and (C) 1.7 on the extent to which the boundary region is labelled.

Primordia label same

This setting allows for the parent labels of cells marked as belonging to primordia to be given a prefix which allows cells of one primordium to be distinguished from those of another. If this setting is set to Yes then the cells of all primordia that you label will be given the same parent labels.
If this setting is set as No then a prefix will automatically be placed in front of the parent labels. The prefix is applied in increments of 2000; so a cell belonging to second layer of the first primordium you mark will carry the parent label 2012 whilst a cell in the boundary region of the third primordium will be labelled 6009.
In the current edition of Cell Atlas 3D, the prefixes are automatically generated meaning that they cannot be customized. However, because MGX is programmed to use 16 colours, prefixes must be a multiple of 16 (hence why increments of 2000 are hard-coded) to preserve the conventional colour scheme. Users should bear this in mind if future editions Cell Atlas 3D allow customisable prefixes.

Step 5. Process output format.
After labelling is finished, Cell Atlas 3D will have assigned parent labels to cells. The labels correspond to these colours/cell positions. (x) denotes an optional prefix that may be added to parent labels when labelling primordia:

1 – First layer
2 – Second layer
3 – Third layer
4 – WUSCHEL domain
5 – First layer, organizing center
6 – Second layer, organizing center
7 – Third layer, organizing center
8 – N/A
(x)9 – Boundary region
10 –N/A
(x)11 – First layer, primordia
(x)12 – Second layer, primordia
(x)13 – Third layer, primordia
